# A salting out and resin procedure for extracting *Schistosoma mansoni *DNA from human urine samples

**DOI:** 10.1186/1756-0500-3-115

**Published:** 2010-04-26

**Authors:** Martin J Enk, Guilherme Oliveira e Silva, Nilton B Rodrigues

**Affiliations:** 1 Laboratório de Esquistossomose - Centro de Pesquisas René Rachou (CPqRR) - Fundação Oswaldo Cruz (FIOCRUZ), Av. Augusto de Lima 1715, Belo Horizonte, Minas Gerais, 30190-002, Brazil; 2 Laboratório de Imunologia Celular e Molecular - Centro de Pesquisas René Rachou (CPqRR) - Fundação Oswaldo Cruz (FIOCRUZ), Av. Augusto de Lima 1715, Belo Horizonte, Minas Gerais, 30190-002, Brazil; 3 Laboratório de Pesquisas Clínicas - Escola de Farmácia, Universidade Federal de Ouro Preto (UFOP), Campus Morro de Cruzeiro, Ouro Preto, Minas Gerais, 35400-00, Brazil

## Abstract

**Background:**

In this paper a simple and cheap salting out and resin (InstaGene matrix^® ^resin - BioRad) DNA extraction method from urine for PCR assays is introduced. The DNA of the fluke *Schistosoma mansoni *was chosen as the target since schistosomiasis lacks a suitable diagnostic tool which is sensitive enough to detect low worm burden. It is well known that the PCR technique provides high sensitivity and specificity in detecting parasite DNA. Therefore it is of paramount importance to take advantage of its excellent performance by providing a simple to handle and reliable DNA extraction procedure, which permits the diagnosis of the disease in easily obtainable urine samples.

**Findings:**

The description of the extraction procedure is given. This extraction procedure was tested for reproducibility and efficiency in artificially contaminated human urine samples. The reproducibility reached 100%, showing positive results in 5 assay repetitions of 5 tested samples each containing 20 ng DNA/5 ml. The efficiency of the extraction procedure was also evaluated in a serial dilution of the original 20 ng DNA/5 ml sample. Detectable DNA was extracted when it was at a concentration of 1.28 pg DNA/mL, revealing the high efficiency of this procedure.

**Conclusions:**

This methodology represents a promising tool for schistosomiasis diagnosis utilizing a bio-molecular technique in urine samples which is now ready to be tested under field conditions and may be applicable to the diagnosis of other parasitic diseases.

## Introduction

Schistosomiasis caused by *Schistosoma mansoni *is a major public health problem in countries of Latin America, the Caribbean and Africa [1,2]. Routinely the diagnosis of this disease is based on the detection of parasite eggs in stool. This approach is relatively inexpensive and easy to perform, and provides basic information on prevalence and infection intensity. However, a well known limitation of these coproscopic methods is their lack of sensitivity, especially in low endemic areas and among individual infections with low parasite load [3-5]. In order to overcome this shortcoming multiple sampling and the examination of a larger amount of faeces are necessary, which increases costs considerably, making these techniques too cumbersome for accurate diagnosis under such conditions. Besides this intrinsic limitation of coproscopic stool examinations, the positive effect of successful control programs and the rising numbers of infected travelers and migrants urgently require more sensitive methods for diagnosing infection with *Schistosoma mansoni *[6-8].

As an alternative, serological tests for antibody detection can be applied for the diagnosis of schistosomiasis [9,10]. Unfortunately serological methods seem to have low sensitivity, cross-reaction with other helmith infections and poor performance in distinguishing between active and past infections, which is particularly important for endemic areas. Furthermore these techniques require collection of blood, an invasive procedure which presents another disadvantage for their application in a large scale [11].

PCR-based diagnostic techniques have shown high sensitivity and specificity, and rely on the detection of *S. mansoni *DNA in feces, serum [12-14] and, recently, in plasma [15] and urine [16]. The use of urine as source of DNA in PCR detection of parasites has been already reported for *Borrelia burgdorferi *[17], *Wuchereria bancrofti *[18], *Mycobacterium tuberculosis *[19,20] and *Schistosoma *sp. [16]. In all cases the extraction method relies on the use of organic solvents, such as phenol and chloroform, or commercial kits that make the process hazardous and/or expensive to use when there are a large number of samples. Here we present a simple salting out and resin DNA extraction method for PCR.

## Methods

Fifty milliliters of fresh urine, of a non infected individual, were treated with EDTA to a final concentration of 40 mM [21]. To assess the reproducibility of the extraction method, 30 milliliters of this sample were artificially contaminated with 120 ng of adult *S. mansoni *DNA and divided in 6 aliquots of 5 ml each, containing 20 ng *S. mansoni *DNA per aliquot, equivalent 4 ng DNA/mL. Five of these aliquots, forming the 1^st ^set of samples, were directly processed. To test the method's efficiency, the sixth aliquot was serially diluted in five consecutive 1:5 dilutions, in 4 mL of the remaining 20 mL non contaminated urine, forming a 2^nd ^set of 6 samples. The DNA concentration for each of these samples was 4 ng/mL, 800 pg/mL, 160 pg/mL, 32 pg/mL, 6,40 pg/mL and 1.28 pg/mL.

All 11 aliquots prepared as described above, were heated at 100°C, in a water bath for 10 min. After that, 5 *M *NaCl, in a volume of 1/10 of the sample volume was added to each tube. The tubes were shaken vigorously for 15 sec, placed on ice for 1 hr and centrifuged for 10 min at 4,000 rpm. The supernatant was transferred to another tube, shaken vigorously for 15 sec and centrifuged for 10 min at 4,000 rpm. Again the supernatant was transferred to another clean tube, and two times the sample volume of absolute ethanol was added. The DNA was then precipitated at -20°C for at least 2 hr. The DNA strand was removed with a pipette, transferred to a 0,5 mL microcentrifuge tube and washed in 200 μL 70% ethanol. The tubes were centrifuged again for 20 min at 14,000 rpm. The pellets were dried and resuspended in 100 μL of DNAse free water and 100 μL of InstaGene matrix^® ^resin (BioRad). Samples were incubated at 56°C for 30 min and 100°C for 8 min, vortexed at high speed for 10 sec and centrifuged at 14,000 rpm for 3 min. The supernatant transferred to a new tube and used as template in PCR reactions.

PCR was carried out using a pair of primers, directed to a 121 bp repetitive fragment, designed by Pontes et al. 2002 [12], GoTaq DNA polymerase and STR 10× buffer (Promega). Into each reaction tube were added 1 μL of 10× buffer, 0.1 μg/μL of 1× BSA, 0.8 U of Taq DNA polymerase, 0.5 pmol of each, forward and reverse primers, 4 μL of DNA template and enough water to a final volume of 10 μL. A positive and negative control were performed using 1 ng of *S. mansoni *DNA as template for the positive, and 1 μL of non-contaminated urine as template for the negative. A total of 40 cycles of amplification were conducted using a 30 sec. denaturing step at 95°C, 30 sec. annealing 65°C and 30 sec. extension at 72°C. PCR assays were conducted 5 times for each of the 11 DNA samples. Three μL of amplified products were visualized by electrophoresis in 8% polyacrylamide gel (PAGE) followed by silver staining [22] and recorded by digital photography.

The study objectives were explained to the participant and informed written consent was obtained in compliance with the guidelines of the Helsinki Declaration about research carried out on humans. The study protocol was received and approved by the Ethical Review Board of the René Rachou Research Center/Fiocruz (No. 03/2008) as well as by the Brazilian Ethical Review Board (CONEP - No 784/2008).

## Results and Discussion

**Figure 1:**
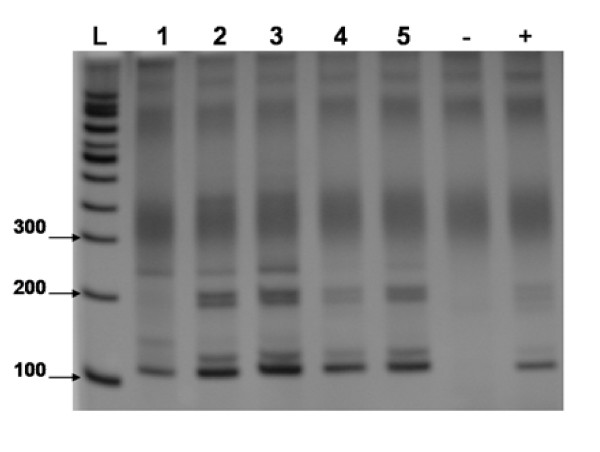
**An 8% polyacrylamide gel, representative of 5 assays, showing the reproducibility test of the extraction method**. L = 100 bp Ladder, 1-5 = 1^st ^sample set 4 ng/mL DNA. - = negative control. + = *S. mansoni *DNA positive control.

**Figure 2:**
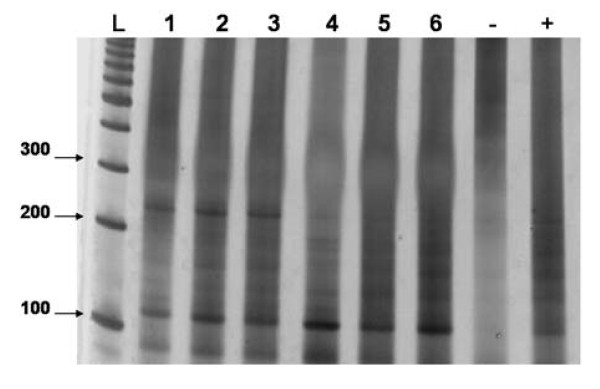
**An 8% polyacrylamide gel, representative of 5 assays, showing the efficiency test of the extraction method**. L = 100 bp Ladder, 1-6 = 2^nd ^sample set, 4 ng/mL, 800 pg/mL, 160 pg/mL, 32 pg/mL, 6,4 pg/mL and 1.28 pg/mL of DNA respectively. - = negative control. + = *S. mansoni *DNA positive control.

Positive results were observed for all 5 samples from the 1^st ^set (figure 1), as well as for all 6 samples from 2^nd ^set (figure 2) in all of the 5 assay repetitions. The results from the 1^st ^sample set show the high reproducibility of the DNA extraction method, and those from the 2^nd ^confirm the test's efficiency by detecting 1.28 pg DNA/mL urine, an approximately 3,000 times smaller quantity of DNA than in the first dilution (results samples set 2). Furthermore, these data confirm the PCR reproducibility and sensitivity, hence, parasite DNA was detected in all 5 assay repetitions.

This simple and inexpensive extraction method, utilizing easy accessible urine samples as DNA source, in combination with the high sensitivity of PCR, constitutes a promising diagnostic tool to overcome the difficulties of detecting schistosomiasis infections in areas of low transmission or in individual cases with low worm burden. As the *S. mansoni *genome contains approximately 580 fg of DNA [23] our proposed method detects theoretically the equivalent of two parasite cells in form of diluted DNA in urine. The detection limit of 0,1 pg parasite DNA of the described technique is comparable to those of other studies carried out in human and non-human samples of feces, urine and blood which range between 1 fg and 1 pg. Pontes et al 2002 [12] reports 1 fg in human feces, Sandoval et al 2006 [16] 1 fg in human urine, Wichmann et al 2009 [15] 2 parasite DNA copies/mL (about 1 pg) in human serum, Kato-Hayashi et al 2010 [24] 0,1 pg in non-human urine and serum and Akinwale et al 2008 [25] 10 fg in diluted parasite DNA.

The use of nontoxic materials during the extraction process and its easy handling, may, in future, make this technique applicable under conditions in which there are limited resources. The data obtained by this study provide sufficient evidence to test this technique in urine samples collected from the field, especially from low prevalence areas, in order to define sensitivity and specificity in comparison to diagnostic tests currently in use. After adaptation, the procedure described here could, also be applied for the detection of trans-renal and/or cell-free DNA of other parasites as well as for viral, bacterial and fungal infections in future studies.

## Competing interests

The authors declare that they have no competing interests.

## Authors' contributions

MJE, and NBR designed the study protocol; MJE, GOS and NBR supervised and carried out the laboratory work and data collection; MJE, GOS and NBR carried out the analysis and interpretation of these data. MJE, GOS and NBR drafted the manuscript. All authors read and approved the final manuscript.
